# Structural Analysis of Cytochrome P450 105N1 Involved in the Biosynthesis of the Zincophore, Coelibactin

**DOI:** 10.3390/ijms13078500

**Published:** 2012-06-01

**Authors:** Bin Zhao, Suzy C. Moody, Robert C. Hider, Li Lei, Steven L. Kelly, Michael R. Waterman, David C. Lamb

**Affiliations:** 1 Department of Biochemistry, School of Medicine, Vanderbilt University, Nashville, TN 37232, USA; E-Mails: li.lei@vanderbilt.edu (L.L.); michael.waterman@vanderbilt.edu (M.R.W.); 2 Institute of Life Science, Medical School, Swansea University, Singleton Park, Swansea, SA2 8PP, UK; E-Mails: s.moody.562842@swansea.ac.uk (S.C.M.); s.l.kelly@swansea.ac.uk (S.L.K.); 3 Institute of Pharmaceutical Science, King’s College London, 5th Floor, Franklin-Wilkins Building, 150 Stamford Street, London, SE1 9NH, UK; E-Mail: robert.hider@kcl.ac.uk

**Keywords:** cytochrome P450, CYP105N1, siderophore, *Streptomyces coelicolor* A3(2), zinc chelation

## Abstract

Coelibactin is a putative non-ribosomally synthesized peptide with predicted zincophore activity and which has been implicated in antibiotic regulation in *Streptomyces coelicolor* A3(2). The coelibactin biosynthetic pathway contains a stereo- and regio-specific monooxygenation step catalyzed by a cytochrome P450 enzyme (CYP105N1). We have determined the X-ray crystal structure of CYP105N1 at 2.9 Å and analyzed it in the context of the bacterial CYP105 family as a whole. The crystal structure reveals a channel between the α-helical domain and the β-sheet domain exposing the heme pocket and the long helix I to the solvent. This wide-open conformation of CYP105N1 may be related to the bulky substrate coelibactin. The ligand-free CYP105N1 structure has enough room in the substrate access channel to allow the coelibactin to enter into the active site. Analysis of typical siderophore ligands suggests that CYP105N1 may produce derivatives of coelibactin, which would then be able to chelate the zinc divalent cation.

## 1. Introduction

Cytochrome P450 monooxygenases (CYP or P450) constitute a superfamily of heme-containing enzymes with more than 16,000 known genes distributed among all biological kingdoms [1]. CYP proteins have diverse primary amino acid sequences and are grouped into different families when they have less than 40% amino acid sequence identity and into sub-families of the same family when they have more than 55% amino acid sequence identity [2]. CYP enzymes are involved both in endogenous biosynthetic pathways e.g., sterol, steroid, vitamin and hormone biosynthesis and in the metabolism of xenobiotic compounds [3]. Enzymatically, CYP catalyzes the molecular scission of atmospheric dioxygen, generally introducing one oxygen atom into a non-activated carbon-hydrogen bond of the substrate with an exceptionally high degree of regio- and stereo-selectivity, the second oxygen atom reduced to a water molecule [4,5]. Basic CYP enzymatic reactions include C-hydroxylation, heteroatom oxygenation, heteroatom release (dealkylation), epoxide formation and group migration. Recently, more complex enzymatic reactions including chlorine oxygenation, dimer formation, ring coupling, ring formation, ring contraction and aromatic dehalogenation have also been attributed as being CYP-catalyzed [6]. Nearly all CYPs use NAD(P)H as a source of electrons for the activation of dioxygen and a redox partner(s) which delivers the electrons to the iron atom of the CYP prothetic heme (generally NAD(P)H cytcohrome P450 reductase for eukaryotic CYPs and combination of a ferredoxin and a ferredoxin reductase for prokaryotic CYPs) [7].

Although most bacteria have no CYPs encoded within their genomes, the actinomycetes have provided a rich source of *CYP* sequences [8]. The genus *Streptomyces* are distinguished by their ability to produce a wide array of biologically active secondary metabolites, many of which have been applied as antibiotics in medicine [9,10]. Additionally, many of these secondary metabolite biosynthetic pathways have one or more cytochrome P450 catalyzed steps which are essential in producing the final pharmacologically active molecule [11]. By far the most prevalent CYP families in *Streptomyces* are the CYP105 and the CYP107 families, with members present in each completely sequenced streptomycete genome as well as being found in numerous other *Streptomyces* species [1]. Generally, CYP107s seem to have been recruited into antibiotic biosynthetic pathways where they play roles in oxidative tailoring the final antibiotic molecule. For example, CYP107A1 (EryF), from the actinomycete bacterium *Saccharopolyspora erythraea*, is responsible for the final step of the biosynthesis of the antibiotic erythromycin [12] and CYP107L1 (PikC) from *Streptomyces venezulae* is responsible for the final step in producing the active antibiotic picromycin [13]. In contrast, CYP105 enzymes are seemingly more promiscuous in their range of catalytic activities with some members having a diverse range of substrates such as CYP105D1, which has been shown to be a xenobiotic metabolising P450 [14], while other members such as CYP105P1 have strict substrate specificity where it is involved in the biosynthesis of the polyene antifungal agent filipin [15].

*Streptomyces* coelicolor A3(2) has been investigated extensively as a model system for the study of morphological and physiological development of *Streptomyces* and for investigation of the genetic control of secondary metabolites [8]. *S. coelicolor* was the first streptomycete to have its genome sequenced and consists of a 8,667,507 bp linear chromosome containing approximately 7825 genes including 20 gene clusters which direct the biosynthesis of known or predicted secondary metabolites [9]. Of the 18 *CYP* gene sequences in *S. coelicolor*, 3 *CYP*s were directly linked to these secondary metabolite gene clusters [9,16]. First, CYP158A2 (SCO1206) as determined by our laboratory, is involved in the oxidative C–C coupling of flaviolin producing diflaviolin, which was then polymerized to melanin-like pigments that are thought to afford protection to the soil dwelling bacterium from the deleterious effects of UV irradiation [17]. Second, CYP170A1 (SCO5223), which we determined as catalyzing two sequential oxidation reactions of the terpenoid *epi*-isozizaene, through two epimers of albaflavenol, to the single sequiterpene antibiotic, albaflavenone [18]. Both the crystal structures of CYP158A2 and CYP170A1 were resolved allowing for detailed mechanistic insight into the catalytic activities [17,19].

**Figure 1:**
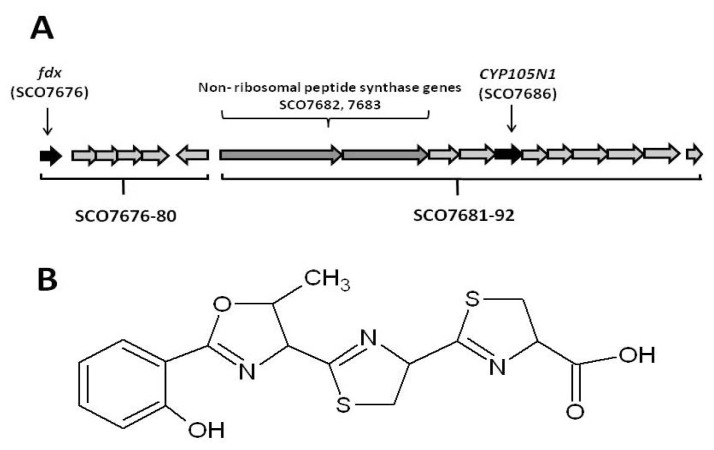
(**A**) The secondary metabolite gene cluster which encodes for the biosynthesis of coelibactin in *Streptomyces coelicolor* A3(2). Genes SCO7676-80 encode a ferredoxin (SCO7676, colored black) and four putative metal transport proteins (SCO7677-80). Genes SCO7681-92 encode the coelibactin biosynthetic pathway including the non-ribosomal peptide synthase genes (SCO7682, 7683, colored grey) which synthesise the initial coelbactin molecule which is then enzymatically tailored by CYP105N1 (SCO7686, colored black) and possibly by proteins of unknown function (SC7684, 7685, 7688 and 7692). SCO7689 and SCO7690 encode putative ABC transporters. (**B**) The predicted structure of coelibactin as determined by analysis of the sequence of the nonribosomal peptide synthetase (NRPS) proteins and prior to enzymatic tailoring.

The final *S. coelicolor* A3(2) CYP unequivocally linked to the biosynthesis of a predicted secondary metabolite in *S. coelicolor* A3(2) is CYP105N1 (SCO7686). *CYP105N1* is one of 17 genes from the gene cluster assigned as being responsible for the production of coelibactin (Figure 1A). Coelibactin is a non-ribosomal peptide derivative whose chemical structure was predicted by computational analyses and from the sequence of its non-ribosomal peptide synthetase (SCO7682, 7683; Figure 1B) [9]. However, coelibactin itself, or any intermediate, has not been identified in *S. coelicolor* A3(2) extracts and hence its chemical structure awaits elucidation. Physiologically, coelibactin has predicted zincophore activity and the expression of the coelibactin gene cluster has been shown to abolish antibiotic production in *S. coelicolor* A3(2) indicating that coelibactin is an important regulator of antibiotic biosynthesis [20,21]. Herein, we present the ligand-free X-ray crystal structure of CYP105N1, which is predicted to be involved in production of the final and biologically active coelibactin molecule. Further, a biochemical reaction mechanism is proposed for CYP105N1, which if accurate, will afford the necessary chemical properties to the coelibactin molecule in order to chelate zinc.

## 2. Results and Discussion

### 2.1. The Role of the Coelibactin Operon in Zinc Chelation and Bioinformatics Analysis

Recently, two reports have emerged in the literature which have implicated the coelibactin gene cluster in indirectly regulating antibiotic production via its role as a zincophore [20,21]. Zinc is a common element in soil and the second most abundant transition metal in cells after iron. Many proteins utilize Zn structurally and if it is allowed to accumulate intracellularly, it is toxic. Previously, intracellular Zn levels were thought to be regulated in bacteria by transport proteins. However, two regulatory mutants of *S. coelicolor* A3(2) (*zur* and *absC*) were characterized and experimental evidence suggests they work by inhibiting expression of the coelibactin gene cluster. In a complicated scenario, the intracellular concentration of zinc directly affects the function of both of these repressor proteins on the expression of the coelibactin gene cluster, suggesting that coelibactin putatively functions by controlling the intracellular concentrations of zinc in *S.* coelicolor A3(2). In the absence of Zur and AbsC, the coelibactin pathway is switched on and antibiotic biosynthesis is lost. Conversely, in the presence of Zur and AbsC, the coelibactin pathway is switched off and antibiotic production can occur. Bioinformatic analysis of both the NRPS (nonribosomal peptide synthetase) proteins reveals that putative homologues of this pathway exist in other streptomycetes. However production of other “coelibactin-like” molecules in other organisms does not require a CYP105N1 homologue in the biosynthetic pathway. For example, in *Salinispora tropica*, a gene encoding a CYP105N1 homologue is missing in its putative zincophore biosynthetic pathway, but two other P450 enzymes are present—CYP1000A1 (Strop_2825) and CYP1000B1 (Strop_2829)—Which constitute two new P450 families with no homology to any existing P450 member. Clearly, the final ‘coelibactin’-structural moiety is different dependent upon the actinomycete in question with the differences possibly at the level of the tailoring of the final molecule. In an effort to elucidate both the final coelibactin molecule and the putative CYP105N1 substrate, we undertook a metabolomic approach by examining both the Zur mutant in which the coelibactin gene cluster was overexpressed and by incubating metabolite extracts with CYP105N1 to trap a potential substrate. No promising candidates were observed in extract analysis. Other laboratories have also tried to identify the product of the coelibactin biosynthetic cluster but with no success (M. Bibb, personal communication).

### 2.2. Bioinformatic Analysis of CYP105N1

The CYP105 family of the P450 superfamily of enzymes is one of the most widespread and studied P450 families amongst bacteria and to-date all sequenced streptomycete genomes have at least one member [1]. Many other CYP105s in streptomycetes were identified through antibiotic gene cluster analysis, through their identification as being involved in key industrial biotransformations e.g., CYP105A3 from *Streptomyces carbophilus* involved in the conversion of ML-236B to pravastatin, a major cholesterol lowering drug through inhibition of HMG-CoA reductase [22], and those involved in drug metabolite production for toxicology testing e.g., CYP105D1 from *Streptomyces griseus* which has a diverse substrate range in the xenobiotics which it can metabolize [14]. Consequently, the chemical substrate range for CYP105 members is widespread and diverse. Given their obvious importance much interest has focused on the structure/function of CYP105 P450s. Hence, the crystal structure of 5 CYP105 members with diverse catalytic activities have been solved: CYP105A1 involved in vitamin D3 metabolism [23]; CYP105A3, involved in the biosynthesis of pravastatin [24]; moxA (CYP105AB3), involved in xenobiotic degradation [25]; and CYP105P1 and CYP105D6 involved in the production of the antifungal agent filipin [15]. CYP105N1, was recently identified in *S. coelicolor* A3(2) as belonging to a gene cluster described herein.

### 2.3. CYP105N1 Expression, Purification and Spectral Analysis

**Figure 2:**
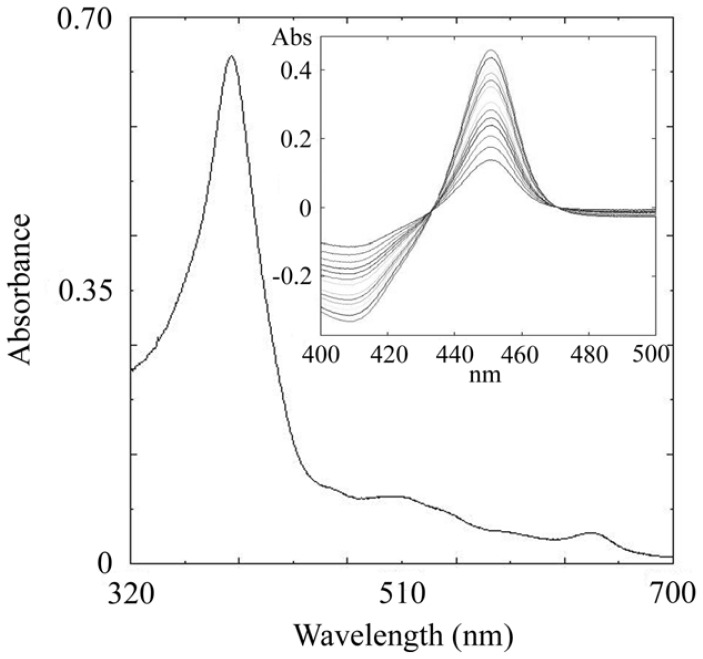
Absolute and carbon monoxide difference spectra of CYP105N1. Absorption spectrum of CYP105N1 (1 μM) in the oxidized (ferric) state and (*inset*) the reduced carbon monoxide difference spectrum of CYP105N1 (1 μM) showing a Soret maximum at 450 nm.

Similarly to the previous expression of some *S. coelicolor* A3(2) P450s, CYP105N1 produced negligible levels of correctly folded P450 protein when expressed directly in *E. coli* under the control of the lac promoter in pET17b (Novagen). Correctly folded CYP105N1 was produced by co-expressing it with the molecular chaperones GroES and GroEL that have been shown to enhance the production of a range of active and correctly folded P450s [26]. In the presence of these proteins expression of active CYP105N1 was greatly enhanced with levels of P450 reaching > 900 nmol P450/L culture after 72 h culture. Cell fractionation revealed CYP105N1 to be a soluble protein being located in the cytosolic fraction following ultracentrifugation at 100,000 × *g*. No P450 was detected in the isolated *E. coli* membranes. After two consecutive Ni^2+^-affinity chromatography purification steps a homogeneous band was observed on sodium dodecyl sulfate-polyacrylamide gel electrophoresis (SDS-PAGE) at approximately 50 kDa compared with the predicted molecular mass of the protein (49.2 kDa). Purified CYP105N1 had a Soret (γ) maximum at 419.2 nm and virtually equal intensities for the distinguishable α – (569 nm) and β – (535 nm) indicating that the P450 is in the oxidized enzymatically active form with the majority of the heme iron in the low spin state (Figure 2). Additionally, purified CYP105N1 protein has a typical P450-reduced CO spectrum, with the spectral maximum at 448 nm (Figure 2, inset).

### 2.4. Features of the Crystal Structure of CYP105N1

#### 2.4.1. The Overall Structure of CYP105N1

**Figure 3:**
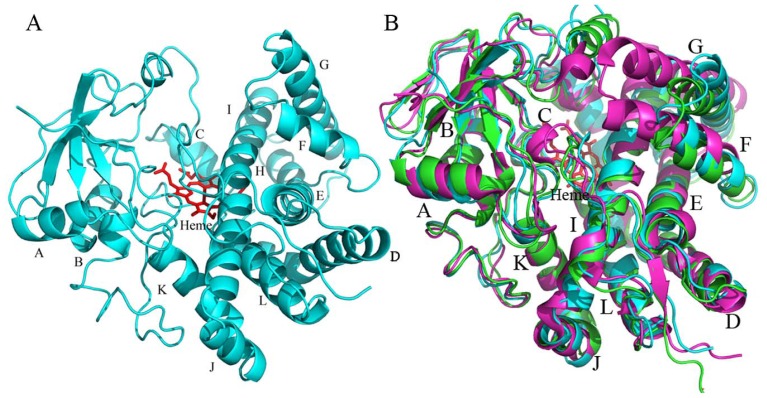
Ribbon diagram of CYP105N1. (**A**) The X-ray crystal structures show a typical cytochrome P450 fold. The ligand-free structure is represented in cyan, heme is shown as a red stick model; (**B**) Overlaid ribbon diagrams of CYP105N1 (cyan), CYP105AB3 ligand-free (green) and CYP105A1 imidazole-bound (magenta) structures.

The crystal structure of CYP105N1 was determined by molecular replacement and was refined at a maximum resolution of 2.9 Å. The final model is defined in the electron density from Pro17 through Trp411 with two or three His412-414 (the residues of the 4 × C-terminal His tag). The overall structure of the CYP105N1 exhibits the typical P450-fold consisting of α-helical and β sheet domains as seen in all other known P450 structures (Figure 3). In detail, the tertiary structure consists of 12 major α-helices and three-group of β-sheets in a triangular shape. The heme cofactor is located between the α-helical domain and the β-sheet domain to create a substrate-binding pocket. The only conserved amino acid (Cys360 in CYP105N1) in all known CYP sequences serves as the fifth axial thiolate ligand to the heme iron. There are four CYP105N1 molecules in the asymmetric unit cell. The secondary structural elements in each of the four structures are almost identical and adopt similar conformations with the root-mean-square deviation (RMSD) between 0.5–0.7 Å in all C_α_ atoms. The slight differences among them were found in the BC loop and FG loop regions, which are the dynamic structural elements in most P450s.

The interesting feature of the CYP105N1 structure is that a large cleft between the α-helical domain and the β-sheet domain exits in all four CYP105N1 structures indicating that the ligand-free CYP105N1 structure is in a wide-open conformation. This opening on the distal surface exposes the heme pocket and the long helix I to the solvent so that the substrate could easily access the active site. This cleft is about 20 Å wide and is formed primarily by the FG loop and helices F and G on the right side and by portions of the BC-loop and the B′ helix on the left side. The cleft results in the curved loop between the helices B and C. Also, the F and G helices swing away from the active site. Such a large opening in the ligand-free CYP105N1 structure might be consistent with the bulky endogenous substrate ceolibactin. Another structural feature of CYP105N1 is that it does not have the B′ helix in the BC-loop region, which is present in many other CYP structures such as CYP105AB3. Instead of the B′ helix, a coiled loop structure is present in this position in CYP105N1.

#### 2.4.2. The Heme-Binding Pocket

The I-helix, the longest α-helix in P450 structures, runs across the entire molecule and above the heme. Several residues in the I-helix may play important roles in proton transfer during catalysis or providing substrate interaction. For example, the hydroxyl group of the Thr253 in the central I helix points toward the heme pocket. This threonine residue is highly conserved in the majority of P450s and may be involved in stabilizing the dioxygen complex and assist in proton transfer. The Ile245 and Ala249 faced to the active site may create hydrophobic environments and van der Waals interactions. Several other hydrophobic residues over the heme could help to form the substrate-binding pocket, such as Pro98, Ile100, Leu198, Ile299, and Ala400. The side chains of Arg95, Arg101, Asn188, and Asp398 are oriented toward the active site pocket of the enzyme as well, which may create a hydrogen-bonding interaction with the substrate. Thus, CYP105N1 exhibits a large cavity proximal to the heme and accommodates the hypothetical interaction with coelibactin.

#### 2.4.3. Comparison of CYP105N1 with Other CYP105s

There are 77 family members in 32 CYP105 subfamilies identified in the CYP105 family [27]. Although few of CYP105 enzyme activities have been characterized [15,23–25], these results suggest that the CYP105 family has broad substrate specificity involved in xenobiotic oxidation as well as secondary metabolite biosynthesis. To date, there are five CYP105 subfamily structures in PDB: CYP105A1, CYP105AB3, CYP105P1, CYP105D6 and CYP105N1.

While CYP105N1 contains about 40% amino acid sequence identity to CYP105AB3, the overall structures of the ligand-free forms including BC-loop and FG region are very similar (rmsd, root mean square deviation, 1.7 Å for all Cα atoms) (Figure 3B). Most secondary structural elements such as helices I and L also superimpose quite well, however, the BC-loop, C helix and FG region show significant differences. There is no α helical structure in BC-loop in CYP105N1, however a B′ helix is present in CYP105AB3. The BC-loop in CYP105N1 is slightly away from the active site compared with that in CYP105AB3. The N-terminus of the C helix in CYP105AB3 is bent more toward the solvent surface than that in CYP105N1. The F helix in both CYP105N1 and CYP105AB3 has the same length and the same α helical turn, however, the C-terminus of the helix G in CYP105AB3 is bent toward the F helix so that the FG-loop is repositioned. All these observed differences between CYP105N1 and CYP105AB3 may suggest the substrate specificity and recognition.

CYP105N1 has 46% amino acid sequence identity to CYP105A1, which is known to convert Vitamin D_3_ to 25-hydroxyvitamin D_3_. CYP105A1 structure (PDB: 2ZBX) shows typical P450 “closed” conformation due to imidazole binding, which increases the rmsd to 2.7 Å for all Cα atoms (Figure 3B). The BC loop is stretching to occupy the partial space of the cleft and the helices F and G rotate into the active site. Thus, both the BC loop and the FG loop are closer in order to close the substrate access channel. The N terminus of helix I exhibits a small bend over the heme group compared with the straight I helix in CYP105N1. These observed differences might indicate CYP105N1 would experience similar secondary structural elements movements when the enzyme interacts with the substrate. The ligand-free structures of CYP105P1 and CYP105D6 were compared with CYP105N1 structure as well. Because the His72 contacts with heme in CYP105P1, the BC-loop region is deep into the active site, which leads to a quite different BC-loop structure between them. In CYP105D6, there are some disorders in BC-loop and FG-loop, but the remaining structures were overlaid well with CYP105N1. This may suggest that CYP105N1 might adopt a large substrate as CYP105D6 does.

### 2.5. Conjecture on the Enzymatic Role(s) of CYP105N1 in Production of an Active Siderophore

**Figure 4:**
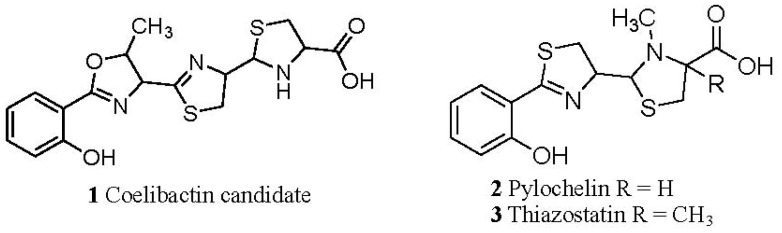
Predicted structure of a possible and active coelibactin zincophore molecule. The predicted structure of coelibactin described by Bentley *et al*. [9], will not satisfy the coordination requirements to form a complex with zinc(II). Reduction of the terminal ring of the structure will generate a coelibactin candidate (**1**) which will bind zinc under physiological conditions in tridentate mode, utilizing the terminal iminoacid and the nitrogen of the adjacent heterocyclic ring (**2**). Without reduction, tridentate coordination is not possible.

The predicted structure for coelibactin as determined by the analysis of the NRPS proteins prior to enzymatic tailoring is given in Figure 1B. Based on analysis of known siderophore structures this molecule is not predicted to possess a high affinity for iron(III) [28]. Furthermore it will not satisfy the coordination requirements of zinc(II). Thus enzymatic modification of this structure must occur in order for it to bind zinc with high affinity. Hydroxylation of the aromatic nucleus or any of the three heterocyclic rings will not create a zinc binding site in the molecule. However reduction of the terminal ring of the structure in Figure 1B generates a coelibactin candidate (1, Figure 4) which will bind zinc under physiological conditions in a tridentate mode, utilizing the terminal imino acid and the nitrogen of the adjacent heterocyclic ring. Without reduction, the tridentate coordination is not possible. Significantly, two analogous siderophores exist, each with the terminal heterocyclic ring being reduced, pyochelin (2) [29] and thiazostatin (3) [30]. Whereas pyochelin chelates both iron(III) and zinc, coelibactin cannot bind iron(III) tightly, as the carboxylate function is too far separated from the phenol group. Thus, coelibactin is predicted to be selective for zinc.

## 3. Experimental Section

### 3.1. Cloning, Heterologous Expression and Purification of *Streptomyces coelicolor* A3(2) CYP105N1

Genomic DNA from *S. coelicolor* A3(2) was isolated according to previously described procedures [9]. Primers 105N1F (5′-GCGCATATGAGCGCCGAATCCACCA-3′) and 105N1R (5′-GCGAAGCTTTCA**ATGGTGATGGTG**GCGGCTCTCGACCCGGAA-3′) were designed to amplify the *S. coelicolor* A3(2) *CYP105N1* ORF. The primers incorporate unique *Nde*I (underlined) and *Hin*dIII (double underlined) cloning sites and a C-terminal polyhistidine tag (bold) to allow purification of the expressed protein by affinity chromatography. PCR conditions were initial denaturation at 95 °C for 2 min, followed by 30 cycles of 95 °C for 20 s, 57 °C for 30 s and 68 °C for 4 min, followed by a final extension step at 68 °C for 7 min. The integrity of *CYP105N1* was confirmed by DNA sequencing. CYP105N1 was expressed and purified using similar conditions as previously described [17,18]. To improve production of correctly folded P450 protein, CYP105N1 was co-expressed with molecular chaperones GroES/GroEL [26]. Briefly, co-transformed cells were cultured overnight in Luria Bertani broth containing 100 μg/mL ampicillin and 50 μg/mL kanamycin. After inoculation (1:100) in 3 L of Terrific Broth containing 100 μg/mL ampicillin and 50 μg/mL kanamycin, growth was carried out at 37 °C and 200 rpm for 6 h. Following the addition of 1 mM σ-aminolevulinic acid for heme synthesis, P450 expression was induced by the addition of 1 mM isopropyl-β-D-thiogalactopyranoside, and chaperone expression was induced by addition of arabinose to a final concentration of 4 mg/mL. Cell growth was continued for an additional 72 h at 27 °C and 170 rpm. Subsequently, the cells were harvested by centrifugation and resuspended in lysis buffer (250 mM sucrose, 50 mM Tris HCl, pH 7.4, 0.5 mM EDTA) and incubated with 1 mg/mL lysozyme for 30 min on ice prior to freezing at −80 °C. Cells were broken by freeze-thawing and the cytosolic fractions separated from cell debris and the membrane fraction by ultracentrifugation at 100,000 × *g* for 1 h. The soluble CYP105N1 was purified by metal (Ni^2+^) affinity chromatography (Qiagen) using established methods [17,18].

### 3.2. Crystallization, Data collection and Structure Determination of CYP105N1

Crystals of CYP105N1 were obtained using hanging-drop vapor diffusion, in which 2 μL of a 20 mg/mL protein solution was mixed with an equal volume of 0.1 M HEPES (pH 7.0), 0.2 M sodium formate, and 20% PEG 3350 at 20 °C. The plate crystals appeared within a few days. The crystals belong to the hexagonal space group P6_1_ (Table 1). Full diffraction data were collected at 100 K at the Life Science Collaborative Access Team (LS-CAT) beamline at the Advanced Photon Source, Argonne National Laboratory, Argonne, IL. The X-ray data were processed and scaled with the HKL package programs HKL2000 [31].

The structure of the CYP105N1 was determined by molecular replacement using the program PHASER [32] and the CYP105A1 structure (PDB: 2ZBX) as a search model. The initial model was built in COOT [33] and refinement was performed using CNS1.3 [34]. There were four molecules of CYP105N1 in the asymmetric unit. Final refinement statistics are given in Table 1. The coordinates and associated structure factors have been deposited with the Protein Data Bank (accession codes: 3TYW). Figures were generated by Pymol [35].

### 3.3. General Methods

Reduced carbon monoxide (CO) difference spectra for quantification of cytochrome P450 content were measured and calculated according to the method described by Omura and Sato [36]. Unless otherwise stated, all chemicals were supplied by Sigma Chemical Company (Poole, Dorset, United Kingdom). UV-visible absorption spectra of purified P450s were recorded using a Hitachi U-3310 scanning spectrophotometer.

## 4. Conclusions

Herein, we present the structure of CYP105N1, the first structure of an enzyme of the coelibactin biosynthetic pathway to be resolved. Coelibactin is the first proposed bacterial zincophore and expression of the coelibactin gene cluster has been implicated in suppressing antibiotic production in *S. coelicolor* A3(2), suggesting a novel mechanism of antibiotic regulation in this organism. Zinc chelation has taken on significant medical importance due to the fact that in Alzheimer’s disease zinc has been implicated in plaque formation through increased formation of amyloid beta peptide. Consequently, the use of zinc chelators as therapeutic agents in Alzheimer’s disease is of growing interest. Coelibactin, being the first naturally occurring zincophore to be discovered, may represent a novel chemical scaffold for development of new therapeutic agents. Furthermore, homologues of the coelibactin gene cluster have been found in other streptomycetes as well as the marine actinomycete *S. tropica,* suggesting that zincophores may be a more widespread mechanism for regulating secondary metabolism than was previously thought and may also be screened as therapeutic agents. Efforts in our laboratory will continue to identify the chemical structure of the active coelibactin molecule and elucidate the CYP105N1 reaction details.

## Figures and Tables

**Table 1:** Data Collection and Refinement Statistics.

Data Collection Statistics	Substrate-free
Space group	P6_1_
Unit Cell (Å)	a = b = 134.537 c = 230.58
Molecules/asymmetric unit	4
Data resolution (Å)	2.9
Redundancy^a^	16.3 (9.9)
Completeness%^a^	99.8 (99.8)
*I*/σ(*I*)^a^	26.8 (6.1)
*Rmerge*%^a^	7.1 (54.2)
Refinement statistics	
No. of reflections used in refinement	51760
No. of water molecules	100
Protein atoms	12366
Heme atoms	172
Ligand atoms	0
*R_work_*%	28.21
*R_free_*%	30.05
*Rmsd*^b^ in bond lengths (Å)	0.009
*Rmsd* in bond angles (°)	1.2
Ramachandran statistics	
Favored regions (%)	94.9
Allowed regions (%)	3.2
Outerliers (%)	1.9

a Values for the highest resolution shell in parentheses;

b Rmsd, root mean square deviation.
